# Sleep Disturbance and Psychological Profiles of Medical Staff and Non-Medical Staff During the Early Outbreak of COVID-19 in Hubei Province, China

**DOI:** 10.3389/fpsyt.2020.00733

**Published:** 2020-07-22

**Authors:** Wei Wang, Wenqin Song, Zhongyuan Xia, Yuhong He, Linghua Tang, Jiabao Hou, Shaoqing Lei

**Affiliations:** ^1^ Department of Anesthesiology, Renmin Hospital of Wuhan University, Wuhan, China; ^2^ Office of Infection Control, Renmin Hospital of Wuhan University, Wuhan, China

**Keywords:** Coronavirus Disease 2019, Pittsburgh sleep quality index, Hospital Anxiety and Depression Scale, frontline medical workers, Mental health, public health emergency, pandemic

## Abstract

**Objective:**

The outbreak of coronavirus disease 2019 (COVID-19) has considerably burdened the healthcare system in the Hubei Province, the most severely affected region in China. The aim of our study was to assess the psychological effects of COVID-19 epidemic on the healthcare workers in Hubei.

**Methods:**

A total of 2737 healthcare workers were sampled using a two-dimensional code shared online between Mar 4 and Mar 9, 2020. The questionnaires consisted of three elements: baseline characteristics, Pittsburgh Sleep Quality Index (PSQI), and Hospital Anxiety and Depression Scale (HADS). The primary outcome variables were PQSI, anxiety and depression scores of non-medical staff, non-frontline medical staff and frontline medical staff. Binary logistical regression analyses were used to compare between respondents with and without sleep disturbance.

**Results:**

About 61.6% of the respondents reported sleep problems, 22.6% experienced anxiety, and 35% exhibited depressive symptoms. The prevalence of sleep disorders was higher among the frontline healthcare workers compared to the non-frontline and non-medical staff, while anxiety and depression were prevalent in the entire cohort. Logistic regression analysis identified medical occupation, family burden, bereavement, anxiety, and depression as significantly predictive of poor sleep quality.

**Conclusions:**

Frontline medical staff are more vulnerable to sleep disturbances. Psychosocial interventions are needed to help allied healthcare personnel to better respond to COVID-19 and future outbreaks.

## Introduction

The first case of coronavirus disease 2019 (COVID-19) was reported in December 2019 in Wuhan, Hubei province, China. It is caused by the severe acute respiratory syndrome coronavirus 2 (SARS-CoV-2) (1, 2), which is transmitted through respiratory droplets and direct human contact, as well as *via* asymptomatic carriers (3, 4). The epidemic rapidly spread from Hubei to other parts of China since an estimated five million people had travelled from Wuhan during the Spring Festival (5). To curb the transmission of COVID-19, the Chinese government imposed quarantine in Wuhan and Hubei since Jan 23–24, 2020. Only one person from each household is permitted to shop for provisions once every two days, except for medical reasons. Furthermore, 19 provincial-level regions have extended logistical support to different areas of Hubei. According to the National Health Commission of China, over 42,000 healthcare workers in 345 medical assistance teams from across the country are in Hubei to control the epidemic as of Mar 8, 2020 (6).

SARS-CoV-2, also known as 2019 novel coronavirus (2019-nCOV), has mainly infected the 30-79 years age group (7, 8), of which 3.5-3.8% are healthcare workers (8, 9). The frontline medical staff must wear enhanced droplet/airborne personal protective equipment, including fit-tested N95 mask, fluid-resistant gown and eye protector, which puts them at a high risk of physiological and psychological problems (10). There were reports of fatigue, poor sleep, fear, anxiety, depression and even post-traumatic stress symptoms (PTSD) among the frontline healthcare workers during the SARS and Ebola outbreaks (11–13). COVID-19-related-PTSD has been shown to be positively correlated with sleep disturbance, anxiety and depression in Chinese and Italian populations, as well as worsening the quality of life of healthcare workers and patients. (14, 15). The psychological intervention medical team from the Second Xiangya Hospital of Central South University, Hunan Province, China found that several nurses serving COVID-19 patients reported excitability, agitation and insomnia (16). Qiu D et al. conducted a systematic review and meta-analysis which showed that allied medical workers are more vulnerable to sleep disturbances compared to the general population in China (17). A multi-center study conducted from late January to early February, 2020 reported a significant correlation between frontline medical jobs and mental health issues (18). It was not until late February 2020 that the shortage of masks, health equipment, physicians and nurses in the Hubei province was effectively addressed. A positive trend in the epidemic prevention and control has emerged in March 9 (19). There is still uncertainty, nevertheless, whether consideration of early March data changes the conclusions regarding the psychological effect of COVID-19 on medical staff and non-medical staff.

In the present study, we analyzed the sleep quality, and the levels of anxiety and depression among the medical and non-medical workers in Hubei province, the most seriously affected region in China in order to determine the psychological effects of the outbreak.

## Methods

### Study Design

The present study was reviewed and approved by the ethic committee of Renmin Hospital of Wuhan University and registered at the Chinese Clinical Trial Registry (ChiCTR2000030985). An anonymous cross-sectional survey was conducted on the medical staff (also referred to as healthcare workers, composed of doctors and nurses) of multiple hospitals and non-medical staff (students, community workers, volunteers, etc.) in the Hubei Province from March 4 to 9, 2020 using an online questionnaire posted as a two-dimensional code. Eligible participants were men and women aged 18-65 years old who had electronic social software. The subjects were instructed to share their responses on WeChat Moments or *via* Email.

### Questionnaires

Questions regarding demographic information such as age, gender, marital status, education, locations in February, occupation during the epidemic and comorbidities (cardiovascular diseases [categorized into hypertension, diabetes, and coronary heart disease], liver and kidney diseases, etc.), sleep quality, anxiety, and depression were included in the questionnaire.

The Pittsburgh Sleep Quality Index (PSQI) score was devised 30 years ago by Buysse et al. to assess multiple factors related to sleep quality. The questionnaire comprises of 19 individual items in seven domains: subjective sleep quality, sleep latency, sleep duration, habitual sleep efficiency, sleep disturbances, use of sleep medication, and daytime dysfunction. Each domain score ranges from 0 to 3 and the total scores from 0–21, with higher scores indicating lower sleep quality. A total PSQI score greater than 5 indicates poor sleep with a diagnostic sensitivity of 89.6% and specificity of 86.5% (20). In addition, subjects with PSQI>10 are considered bad sleepers (20).

The Hospital Anxiety and Depression Scale (HADS) is a 14-item self-administered questionnaire that consists of 7 items for anxiety scale (HADS-A) and 7 items for depression (HADS-D) (21). Each item is rated on a 4-point Likert scale (0–3) and higher scores indicate greater anxiety and depression severity. A score of 8 or more represents probable case of depression (21).

### Statistical Analysis

Statistical analysis was performed using SPSS Statistics version 16.0 software (IBM). Continuous variables were compared using two-tailed t tests or Mann-Whitney tests depending on data distribution. Chi-square or Yates’ continuity corrected chi-square tests were used to compare categorical variables. Associations between risk factors and poor sleep quality were determined with binary logistic regression analysis. P<0.05 was considered statistically significant.

## Results

### Respondent Characteristics

We received responses from 2,777 participants, and 2,737 (98.6%) completed the questionnaires, of which 20 did not conform to the inclusion criteria, 10 contained invalid information and 706 were from outside Hubei Province. For the remaining 2,001 respondents, the median age was 33 years (IQR, 28–40) and median BMI was 22 (IQR, 20.0–24.0). The overwhelming majority (95.7%) of these respondents had more than 12 years of education. In addition, 1,291 (64.5%) respondents were female, 1,419 (70.9%) were married, 1,213 (60.6%) were aged 18–35 years, 126 (6.3%) had underlying comorbidities, 444 (22.2%) reported shortage of supplies, 301 (15.0%) had a COVID-19 patient in their family, and 39 (1.9%) were confirmed or highly suspected of COVID-19. Only 673 (33.6%) respondents were from Wuhan, and the others (66.4%) from other cities in Hubei Province, China (**Table 1**). In terms of occupation, 1,514 (75.7%) were healthcare workers, including 661 frontline medical professionals. Compared to non-healthcare workers, the healthcare workers were older, female, married, had higher education level, and were more likely to be under pressure of caring for the elderly or children (**Table 1**). Frontline medical workers were older and had higher BMI as compared to the non-frontline workers. More male healthcare workers were in the frontline rather than non-frontline jobs (268[40,5%] vs. 249[29.2%]). Among the frontline healthcare workers, 418 (63.2%) had the burden of caring for the elderly or children, and 73 (11%) had recent COVID-related bereavement. These percentages were higher than that for non-frontline healthcare workers. There were no significant differences in the number of smokers and alcohol use between the frontline and non-frontline healthcare workers (**Table 1**).

**Table 1:** Demographics characteristics of study participant.

	Overall cohort	Medical staff		P^a^ value	Frontline medical workers		P^b^ value
	(n=2 001)	No(n=487)	Yes(n=1 514)		No (n = 853)	Yes (n = 661)	
Age(y)	33(28-40)	31(26-37)	33(29-40)	<0.01	33(29-40)	34(30-40)	0.01
18–35	1 213(60.6)	335(68.8)	878(58.0)	<0.01	515(60.4)	363(54.9)	0.03
36–44	480(24.0)	93(19.1)	387(25.6)	<0.01	199(23.3)	188(28.5)	0.02
45–65	308(15.4)	59(12.1)	249(16.4)	0.02	139(16.3)	110(16.6)	0.86
Gender				0.03			<0.01
Male	710(35.5)	193(39.6)	517(34.1)		249(29.2)	268(40.5)	
Female	1 291(64.5)	294(60.4)	997(65.9)		604(70.8)	393(59.5)	
Marital status							
Unmarried	534(26.7)	194(39.8)	340(22.4)	<0.01	191(22.4)	149(22.5)	0.94
Married	1 419(70.9)	276(56.7)	1 143(75.5)	<0.01	646(75.7)	497(75.2)	0.81
Divorced or Widowed	48(2.4)	17(3.5)	31(2.1)	0.07	16(1.9)	15(2.2)	0.59
Education level				<0.01			0.95
≤12years	86(4.3)	72(14.8)	14(0.9)		8(0.9)	6(0.9)	
>12years	1 915(95.7)	415(85.2)	1 500(99.1)		845(99.1)	655(99.1)	
Smoker	167(8.3)	58(13.0)	109(7.2)	<0.01	53(6.2)	56(8.5)	0.09
Alcohol use	354(17.7)	104(21.4)	250(16.5)	<0.01	128(15.0)	122(18.5)	0.07
BMI	22.0 (20.0-24.0)	21.8 (20.0-24.0)	22.0 (20.1-24.1)	0.45	21.8 (19.9-23.9)	22.3 (20.3-24.2)	0.02
Region				0.03			0.11
Wuhan	673(33.6)	184(37.8)	489(32.3)		261(30.6)	228(34.5)	
Hubei province outside Wuhan	1 328(66.4)	303(62.2)	1 025(67.7)		592(69.4)	433(65.5)	
COVID-19 diagnosed or suspected	39(1.9)	4(0.8)	35(2.3)	0.04	15(1.8)	20(3.0)	0.10
Comorbidities	126(6.3)	24(4.9)	102(6.7)	0.15	50(5.9)	52(7.9)	0.12
Cardiovascular diseases	64(3.2)	9(1.8)	55(3.6)	0.052	27(3.2)	28(4.2)	0.27
liver and kidney diseases	14(0.7)	2(0.4)	12(0.8)	0.57	4(0.5)	8(1.2)	0.19
Others	48(2.4)	13(2.7)	35(2.3)	0.65	19(2.2)	16(2.4)	0.80
Shortage of supplies	444(22.2)	106(21.8)	338(22.3)	0.80	173(20.3)	165(25.0)	0.12
Burden of caring for the elderly or children	1 177(58.8)	265(54.4)	912(60.2)	0.02	494(57.9)	418(63.2)	0.04
Family infected with COVID-19	301(15.0)	75(15.4)	226(14.9)	0.80	126(14.8)	100(15.1)	0.85
COVID-related bereavement	164(8.2)	40(8.2)	124(8.2)	0.99	51(6.0)	73(11.0)	<0.01

BMI, body mass index; COVID-19, Corona Virus Disease 2019.

Continuous variables are presented as median (IQR) (Mann-Whitney test). Categorical variables are expressed as number (percentages) (Chi-square or Yates’ continuity corrected chi-square test). ^a^P values indicate differences between non-medical staff and medical staff. ^b^P values indicate differences between non-frontline and frontline medical workers. P < 0·05 was considered statistically significant.

### Effects of the COVID-19 Epidemic on Sleep, Anxiety, and Depression in Frontline Medical Workers

**Figure 1:**
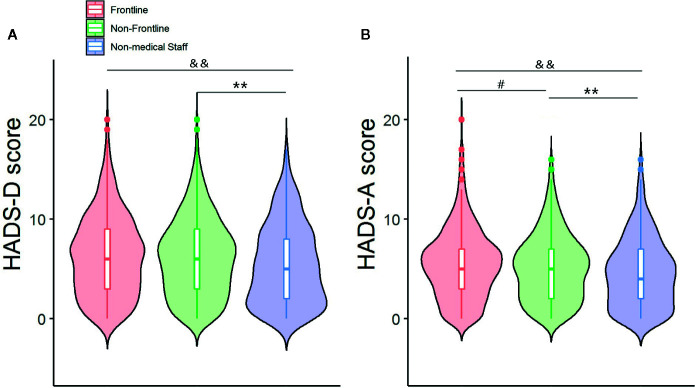
Frontline medical staff mainifested higher HADS-A **(A)** and HADS-D **(B)** scores compared to non-frontline and non-medical staff. Data are presented as median (interquartile range). ^#^p < 0.05, Frontline vs Non-frontline medical staff; **p < 0.01, Non-frontline medical staff vs Non-medical staff; ^&&^p < 0.01 Frontline medical staff vs Non-medical staff.

**Figure 2:**
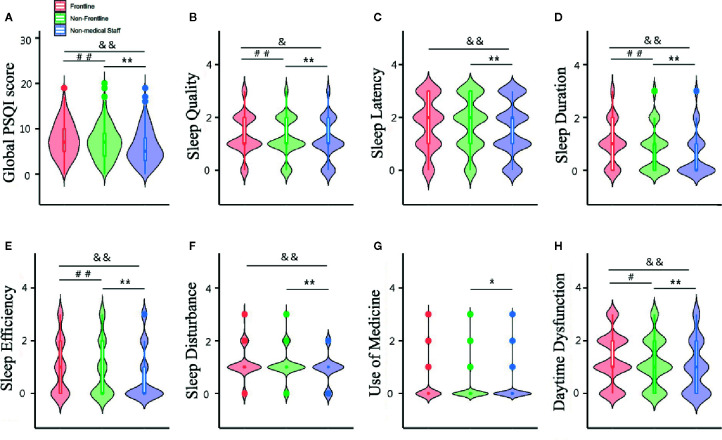
Global PSQI score **(A)** and component scores **(B–H)** of the participants. Data are presented as median (interquartile range). ^##^p < 0.01,^#^p < 0.05, Frontline vs Non-frontline medical staff; **p < 0.01, *p<0.05, Non-frontline medical staff vs Non-medical staff; ^&&^p < 0.01, ^&^p < 0.05, Frontline medical staff vs Non-medical staff.

The median (interquartile range, IQR) PSQI, HADS-A and HADS-D scores of the overall cohort were 7 (4-9), 5 (2-7), and 6 (3-9), respectively. The frontline healthcare workers scored higher for all 3 scales compared to the non-medical staff and non-frontline healthcare workers (**Figures 1** and **2A**). PSQI component scores for sleep quality, sleep duration, habitual sleep efficiency and daytime dysfunction in frontline healthcare workers were significantly higher than that of non-frontline workers and non-medical staff (**Figures 2B, D, E, H**). Medical staff reported higher sleep latency and sleep disturbance scores, compared with non-medical staff (**Figures 2C, F**). There was no significant difference in median component scores of sleep medication use between those working directly with COVID-19 patients and other subgroups (**Figure 2G**). The medical staff were more vulnerable to poor sleep during the COVID-19 epidemic (**Table 2**), while anxiety and depression were prevalent in the entire cohort (**Table 2**). Among the healthcare workers, 1,000 (66.1%) reported poor sleep quality (PSQI>5), of which 301(19.9%) reported very poor sleep quality (PSQI >10). Compared to the non-frontline workers, those in frontline were more likely to experience very poor sleep (156[23.6%] vs 145[17%]). In addition, 354 (23.4%) had anxiety (HADS-A>7), and 546 (36.1%) suffered from depression (HADS-D>7). No significant difference was seen in the prevalence of anxiety or depression between the frontline and non-frontline healthcare workers. (**Table 2**). Medical staff aged 18–35 (comprised of 63.4% and 17.9% of individuals with poor and very poor sleep quality) were more prone to develop sleep disorders than the general public (comprised of 43.0% and 8.7% of poor and very poor sleep quality). Serious sleep problems were more common in young nurses and doctors who had direct contact with infected patients than others in the same age groups (86/363 (35.2%) vs 71/515 [13.8%]) (**Table 2**). Interestingly, the incidence of anxiety and depression in medical staff aged 45-65 were up to 31.3% and 38.6%, respectively, almost twice as much of age-matched non-medical staff (16.9% and 20.3%, respectively) (**Table 2**). However, there was no significant difference in the prevalence of sleep disorders, anxiety or depression between healthcare workers aged 36-44 and their peers (**Table 2**).

**Table 2:** Incidence of poor sleep quality, anxiety, and depression in overall cohort and subgroups.

		Overall cohort (n=2 001)	Medical staff			P^a^ value	Frontline medical workers		P^b^ value
			No(n=487)		Yes(n=1514)		No (n = 853)	Yes (n = 661)	
**Overall age**									
	PSQI>5	1 233 (61.6)	233(47.8)		1 000(66.1)	<0.01	550(64.5)	450(68.1)	0.14
	>10	366 (18.3)	65(13.3)		301(19.9)	<0.01	145(17.0)	156(23.6)	<0.01
	HADS-A>7	452 (22.6)	98(20.1)		354(23.4)	0.13	190(22.3)	164(24.8)	0.25
	HADS-D>7	701 (35.0)	155(31.8)		546(36.1)	0.09	304(35.6)	242(36.6)	0.70
**Age range, y**									
18-35		1 213	335		878		515	363	
	PSQI>5	701(57.8)	144(43.0)		557(63.4)	<0.01	313(60.8)	244 (67.2)	0.051
	>10	186(15.3)	29(8.7)		157(17.9)	<0.01	71(13.8)	86(35.2)	<0.01
	HADS-A>7	234(19.3)	62(18.5)		172(19.6)	0.67	95(18.4)	77(11.6)	0.31
	HADS-D>7	391(32.2)	101(30.1)		290(33.0)	0.34	169(32.8)	121(33.3)	0.87
36-44		480	93		387		199	188	
	PSQI>5	325(67.7)	57(61.3)		268(69.3)	0.14	138(69.3)	130(69.1)	0.97
	>10	102(21.3)	23(24.7)		79(20.4)	0.36	35(17.6)	44(23.4)	0.16
	HADS-A>7	130(27.1)	26(28.0)		104(26.9)	0.83	50(25.1)	54(28.7)	0.42
	HADS-D>7	202(42.1)	42(45.2)		160(41.3)	0.50	77(38.7)	83(44.1)	0.28
45-65		308	59		249		139	110	
	PSQI>5	207(67.2)	32(54.2)		175(70.3)	0.02	99(71.2)	76(69.1)	0.71
	>10	78(25.3)	13(22.0)		65(26.1)	0.52	39(28.1)	26(23.6)	0.43
	HADS-A>7	88(28.6)	10(16.9)		78(31.3)	0.03	45(32.4)	33(30.0)	0.69
	HADS-D>7	108(35.1)	12(20.3)		96(38.6)	<0.01	58(41.7)	38(34.5)	0.25

PSQI, Pittsburgh Sleep Quality Index; HASD-A, Hospital Anxiety and Depression Scale-Anxiety; HASD-D, Hospital Anxiety and Depression Scale-Depression.

Categorical variables are expressed as number (percentages) (Chi-square or Yates’ continuity corrected chi-square test). ^a^P values indicate differences between non-medical staff and medical staff. ^b^P values indicate differences between non-frontline and frontline medical workers. P < 0·05 was considered statistically significant.

### Risk Factors for Poor Sleep Quality

Binary logistic regression showed that male sex, education, smoking, alcohol consumption, BMI, geographical region, and novel coronavirus infection were not predictive of poor sleep quality (**Table 3**). However, age over 35 years, married, comorbidities, medical job, shortage of daily necessities, fostering or parenting pressure, death of a family member, anxiety, and depression were associated with poor sleep quality (**Table 3**). The multivariable analysis indicated that medical personnel, burden of caring for the elderly or children, bereavement, and anxiety-depression were independent risk factors for sleep problems (**Table 3**).

**Table 3:** Binary logistic regression of risk factors for poor sleep quality during COVID-19 outbreak.

Variable	OR (95%CI)			
	UnivariateAnalysis	P value	MultivariableAnalysis	P value
Age (y)				
18-35	Ref			
36-44	1.53 (1.23–1.91)	<0.01	NA	NA
45-65	1.50 (1.15–1.95)	<0.01	NA	NA
Male	0.93 (0.77–1.13)	0.47	NA	NA
Marital status				
Unmarried	Ref			
Married	1.81 (1.48–2.22)	<0.01	NA	NA
Divorced or widowed	1.33 (0.73–2.42)	0.35	NA	NA
Education level		0.37		
≤12years	Ref			
>12years	1.22 (0.79–1.89)		NA	NA
Smoker	1.00 (0.72–1.39)	0.99	NA	NA
Alcohol use	1.12 (0.88–1.42)	0.34	NA	NA
BMI	1.15 (0.96–1.39)			
<18.5	Ref			
18.5-23.9	0.98 (0.69–1.38)	0.89	NA	NA
24.0-30	1.14 (0.79–1.66)	0.48	NA	NA
>30	1.81 (0.68–4.85)	0.24	NA	NA
Region		0.68		
Hubei Outside Wuhan	Ref		NA	NA
Wuhan	1.04 (0.86–1.26)		NA	NA
COVID-19 diagnosed or suspected	1.83 (0.89–3.77)	0.10	NA	NA
Comorbidities	1.90 (1.26–2.87)	<0.01	NA	NA
Occupation				
Non-medical staff	Ref		Ref	
Non-frontline medical staff	1.98 (1.58–2.48)	<0.01	2.07 (1.62–2.64)	<0.01
Frontline medical staff	2.33 (1.83–2.96)	<0.01	2.33 (1.79–3.02)	<0.01
Shortage of supplies	1.51 (1.20–1.89)	<0.01	NA	NA
Burden of caring for the elderly or children	1.76 (1.46–2.11)	<0.01	1.47 (1.18–1.75)	<0.01
Family infected with 2019-nCOV	1.08 (0.84–1.39)	0.56	NA	NA
COVID-related bereavement	2.54 (1.73–3.75)	<0.01	1.91(1.26–2.91)	0.01
Anxiety	5.75 (4.29–7.71)	<0.01	2.98(2.13–4.15)	<0.01
Depression	4.75 (3.80–5.94)	<0.01	2.96(2.30–3.82)	<0.01

BMI, body mass index; COVID-19, Corona Virus Disease 2019; OR, odds ratio; Ref, reference; NA, not applicable.

## Discussion

We analyzed the sleep quality, anxiety and depression in 1,514 medical workers and 487 non-medical workers from Mar 4 to 9, 2020, and found that 61.6% of the respondents had sleep problems, 22.6% felt anxiety, and 35% suffered from depression. Frontline healthcare workers were more prone to severe sleep problems compared to the non-frontline and non-healthcare workers, while no significant differences were seen in the prevalence of anxiety or depression among the three subgroups. Frontline medical staff, non-frontline medical staff, parental burden, COVID-related bereavement, anxiety and depression were the independent risk factors of poor sleep quality.

The COVID-19 outbreak is the third major global public health event caused by coronavirus after SARS and Middle East respiratory syndrome (MERS). Studies show that 21-57% of the medical personnel experienced emotional distress during the SARS outbreak (22–24). Psychiatric morbidity was also observed in almost 51% of the healthcare workers during the MERS outbreak (25). During the early stage of the COVID-19 outbreak, 28.8% of the general population in China reported anxiety symptoms, and 16.5% reported depressive symptoms (26). However, the incidence of anxiety, depression and insomnia among the healthcare workers was 44.6%, 50.4% and 34.0% respectively (18). We found that anxiety, depression and sleep disturbance respectively occurred in 20.1%, 31.8% and 47.8% of the general population, and the incidence was higher among health professionals at 23.4%, 36.1% and 66.1% respectively. In addition, a previous study reported that 39.2% of the medical staff in China suffer from sleep disturbances (17). Nevertheless, positive changes in the COVID-19 situation might alleviate the negative emotions among the healthcare workers in Hubei Province.

The sleep quality was poorer in the frontline medical staff compared to the non-frontline and non-medical cohorts. The mean PSQI scores of SARS vs non-SARS unit nurses at the beginning of the SARS outbreak were 7.2 and 4 respectively (27). A survey on 116 Taiwan nurses showed that sleep quality was worse (average total PSQI score 12) before caring for SARS patients, and continued to be poor (average global PSQI score 8-10) until 3 months afterwards (28). Another survey concluded that mean PSQI scores of SARS unit nurses ranged from 7.2 to 5.2 during a 7-week period (27). Single-center evidence from the early COVID-19 outbreak period indicated that the sleep quality of frontline healthcare personnel was lower with a mean PSQI score of 8.6, compared with normal Chinese residents (29). In our study, the median PSQI scores of both frontline and non-frontline workers were 7, although frontline medical workers were more susceptible to severe sleep disturbances. During the SARS outbreak, 14 to 37% of the frontline staff complained of insomnia compared to 5 to 9.7% of non-frontline workers (27, 30). Bai Y et al. found that the incidence of anxiety-depression was similar between the frontline healthcare workers and administrative staff during a survey conducted from May 29 to June 5, 2003 (28). Consistent with this, anxiety and depression were not significantly different among the three subgroups in our study. This could be attributed to policies for addressing these mental health problems, such as identifying infection as work-related injury, assistance from other provinces, a shift system to allow allied staff to rest, additional salaries or subsidies and psychological intervention (10). Furthermore, the stress due to quarantine, worrying about family members etc. affect both healthcare and non-healthcare workers, and are as significant as medical stressors.

Binary logistic regression analysis identified medical occupation, parental burden, death of a loved one, anxiety and depression as the 5 factors of poor sleep quality. During the SARS outbreak as well, heavy workload, family stress, personal or family lifestyle and negative feelings were significantly correlated with insomnia (23, 27, 31). Studies have also indicated that high anxiety level correlate with sleep quality (32, 33), and a similar association was found in our survey as well. In line with a previous study describing the relationship between depressive symptoms and sleep disturbance (34), allied healthcare workers who suffered from depression were 2.96 (95% CI 2.30–3.82) times more likely to have disturbed sleep compared to those lacking depressive symptoms. Furthermore, sleep problems, especially insomnia, have been bidirectionally implicated in the development of mood disturbances, indicating that insomnia may be a predictor of anxiety and depression, and vice-versa (35). In the case of Chinese physicians, a cross-sectional study concluded that participants who had insufficient sleep (<6 h per day) were more predisposed to anxiety (OR = 2.70, 95% CI: 1.51–4.83) and depression (OR = 1.58, 95% CI: 0.95–2.64) than those who had proper sleep (≥8 h per day) (36). Accordingly, effective amelioration of sleep disorders may help abate the occurrence of subsequent or comorbid mood disturbances and vice-versa (37). There were some limitations in our study that ought to be addressed.

First, an online questionnaire cannot guarantee accurate demographic or other information from the respondents (19). Second, apart from data about shift workers, we did not dynamically observe the psychological impact of COVID-19 outbreak on healthcare professionals. Our survey was conducted when the measures taken for controlling COVID-19 outbreak were showing positive results, which may have affected the psychological status of the respondents since they adapted to coronavirus characterized by high transmissibility but low fatality rates. Third, the questionnaire used for the survey did not include other mental illnesses such as fear, denial, anger and PTSD, since some frontline workers were very reluctant to answer all 57 questions. Moreover, it may be impossible to overcome the selection bias due to the study being based on an online survey. Of the 2,001 respondents in the present study, an overwhelming majority were young females. Ultimately, PSQI was found to be frequently associated with the evaluation of daytime sleepiness therefore it may be useful to add the evaluation of the Epworth sleepiness scale (38). In addition, anxiety and depression cannot be diagnosed by only using questionnaires, therefore further evidence through a psychological evaluation will be required.

## Conclusions

The COVID-19 outbreak has affected the sleep quality of healthcare workers. As the epidemic evolves and psychological interventions are optimized, the physical and mental fitness of the frontline staff may improve. Long-term psychosocial effects of COVID-19 on allied healthcare workers deserve further investigation to prepare for any future public health disasters.

## Data Availability Statement

The original contributions presented in the study are included in the article/supplementary material; further inquiries can be directed to the corresponding authors.

## Ethics Statement

The studies involving human participants were reviewed and approved by: The ethic committee of Renmin Hospital of Wuhan University. Written informed consent to participate in this study was provided by the participants’ legal guardian/next of kin.

## Author Contributions

WW, SW, XZ, HY, and LS designed the study. TL and HJ recruited the respondents. WW and SW collected and analyzed psychological data. WW and SW drafted the manuscript. XZ and LS undertook a critical revision of the manuscript. All authors contributed to the article and approved the submitted version.

## Conflict of Interest

The authors declare that the research was conducted in the absence of any commercial or financial relationships that could be construed as a potential conflict of interest.
